# Association of IL-18 and CXCL10 Levels with Disease Severity in Vietnamese Patients with Dengue Infection

**DOI:** 10.3390/jcm14217753

**Published:** 2025-10-31

**Authors:** Long Hoang Tran, Cuong Duy Do, Phuong Minh Nguyen, Ben Huu Nguyen, Linh Tung Nguyen, Cuong Xuan Hoang, Minh Duc Chu, Su Xuan Hoang

**Affiliations:** 1 Institute of Tropical Medicine, Bach Mai Hospital, Hanoi 115000, Vietnam; thl2@bachmai.edu.vn (L.H.T.); doduy.cuong@gmail.com (C.D.D.); 2 Department of Occupational Medicine, Vietnam Military Medical University, Hanoi 100000, Vietnam; phuongk21@gmail.com (P.M.N.); nguyenben125@gmail.com (B.H.N.); 3 Institute of Health Sciences, Ho Chi Minh City University of Technology, Ho Chi Minh City 72500, Vietnam; linhnt4046@gmail.com; 4 Vietnam Military Medical University, Ho Chi Minh City 700000, Vietnam; hoangxuancuong1605@gmail.com; 5 Institute of Biomedicine and Pharmacy, Vietnam Military Medical University, Hanoi 100000, Vietnam; chuducminh28@gmail.com

**Keywords:** IL-18, CXCL-10, Dengue severity

## Abstract

**Objectives:** to investigate the association of IL-18 and CXCL10 levels with disease severity in Vietnamese patients infected with Dengue virus. **Methods:** A total of 295 serum samples were collected from patients with clinical presentation of Dengue virus infection during the 2022–2023 outbreaks in Hanoi, Vietnam. Clinical and laboratory parameters were recorded at the time of admission. IL-18 and CXCL-10 were measured by standard ELISA assays. The clinical outcome of Dengue infection was classified into three groups according to the WHO 2009 criteria. **Results:** Among the 295 patients, 140 were diagnosed with Dengue without warning signs, 134 with Dengue with warning signs, and 21 with severe Dengue according to the WHO 2009 criteria. Patients with SD had a greater proportion of warning signs than those with DwoWS and DwWS (bleeding: 57.1% versus 47.8% and 1.4%, *p* < 0.001) and (hepatomegaly: 23.8% versus 9.7% and 1.4%, *p* < 0.001). Higher levels of liver enzymes were observed in the SD group than in the DwoWS and DwWS groups (*p* < 0.001). Of note, elevated levels of IL-18 and CXCL-10 levels were found in the SD group compared to the DwoWS and DwWS groups (*p* < 0.01 and *p* = 0.037). Further ROC analysis revealed that the cut-off values for distinguishing severe Dengue from non-severe Dengue for IL-18 and CXCL-10 were both 669.65 pg/mL, whereas that of CXCL-10 was 3739.5 pg/mL. **Conclusions:** The present study showed that IL-18 and CXCL-10 are associated with Dengue severity. This finding suggests that IL-18 and CXCL-10 may be biomarkers for disease progression and disease severity in Dengue infection.

## 1. Introduction

Infection by Dengue virus remains a significant challenge, and unsolved problems in many tropical and subtropical countries influence significant morbidity and mortality [1]. DENV virus belongs to the *Flaviviridae* family and is transmitted by the Aedes mosquito species. There are four distinct serotypes (DENV-1, DENV-2, DENV-3, and DENV-4), and different serotypes circulate in different geographical regions. The clinical spectrum of Dengue infection varies from asymptomatic infection and mild febrile illness to severe Dengue (SD) and death [2]. The pathogenesis of Dengue is a complex interplay between host and viral factors. It has been shown that clinical outcome and disease severity depend on immune responses to each DENV serotype [3]. Individuals infected with one serotype do not have protection against other serotypes, and secondary infection with Dengue may result in more severe illness. DENV infects a variety of cell types, including epithelial cells, endothelial cells, immune cells, and hepatocytes. The activation of immune system contributes to chemokine and cytokine production. However, the excessive release of inflammatory mediators is associated with organ damage and disease progression [3]. There has been substantial evidence suggesting that an increase in cytokine and chemokine levels, such as IFNγ, TNFα, IL-8, IL-18, CXCL-10, in Dengue infection is associated with disease severity [4,5]. The 2009 WHO Dengue guideline recommended the use of warning signs such as early indicators of plasma leakage and organ failure in SD [6]. An important aspect of the disease’s progression to SD commonly occurs after the febrile phase, i.e., between days 4 and 6 of illness. It is essential to identify patients at risk of progressing to SD [7,8]. The potential biomarkers for predicting early Dengue disease severity during the febrile phase therefore play a major role in improving case management and reducing the health-care burden of Dengue in endemic regions. However, it still poses significant challenges for the early diagnosis of severe Dengue in resource-limited settings. Dengue is endemic in Vietnam, with thousands of cases reported per year. Recently, a large outbreak of Dengue fever occurred in Hanoi, with nearly 37.651 cases of Dengue infection and 7 deaths reported during the 2017 outbreak [9]. In this study, we aim to investigate the IL-18 and CXCL-10 levels among adult patients in a tertiary care hospital during the 2022–2023 outbreaks in Hanoi, Vietnam, and to analyze their associations with disease severity.

## 2. Materials and Methods

### 2.1. Patients

We studied 295 patients with suspected Dengue infection who were admitted into the Center of Tropical diseases, Bach Mai Hospital, during the 2022–2023 outbreak in Hanoi, Vietnam. A diagnosis of Dengue infection was defined as patients having positive NS1 Ag alone or IgM antibodies or IgG antibodies using SD BIOLINE Dengue Duo NS1 Ag and the IgG/IgM test (Standard Diagnostic Inc., Yongin-si, Republic of Korea) according to the manufacturer’s instructions. Dengue serotypes were detected by previously published protocols [10]. Clinical data and laboratory parameters were recorded, and clinical outcome were classified according to the WHO’s guidelines as Dengue without warning signs (DwoWS), Dengue with warning signs (DwWS), or severe Dengue (SD) [6]. Plasma samples were collected during the acute phase of Dengue infection (0–6 days) and then stored at −70 °C until use. Patients were given informed consent, and the study protocol was approved by the local ethics committee (7210/QĐ-BM. Date of approval was 31 December 2024).

### 2.2. Quantification of IL-18 and CXCL-10

Quantification of IL-18 and CXCL-10 in the plasma samples was performed using the commercially available Human CXCL10/IP-10 DuoSet ELISA (Catalog #: DY266), and Total IL-18 Duoset ELISA (Catalog #: DY318-05), R&D, Minneapolis, MN, USA. The tests were performed in accordance with the manufacturer’s instructions. All these samples were tested in duplicate. Internal quality control standards were included in each assay, and the standard curve was used for estimation of cytokine concentration (pg/mL).

### 2.3. Statistical Analysis

Statistical analyses were performed with SPSS 20.0 (IBM, Armonk, NY, USA). For categorical variables, a Chi-square test or Fisher’s exact tests were used when appropriate, and Student’s T tests and the Mann–Whitney U test was used for quantitative variables. Correlation of key laboratory parameters with IL-18 and CXCL-10 levels was estimated by Spearman’s correlation. Receiver operating characteristic curve analysis was used to determine the cut-off values of IL-18 and CXCL-10 for predicting severe Dengue. *p* values < 0.05 were considered statistically significant.

## 3. Results

### 3.1. Baseline Characteristics and Laboratory Parameters of Patients with Dengue

In the present study, 295 patients suspected to have Dengue were recruited from the Center of tropical diseases, Bach Mai hospital in Hanoi, Vietnam. Patients were divided into three groups according to the WHO’s guidelines [6]. There were 140 patients with Dengue fever, 134 patients with Dengue with warning signs, and 21 cases of severe Dengue. Clinical and laboratory parameters of the three patient groups are presented in Table 1. There was no significant difference in relation to gender in the three patient groups. The median ages in patients with severe Dengue were higher than the median ages of those in the DwoWS and DwWS groups (58 versus 41.5 and 38 years, *p* < 0.011). Among the clinical manifestations noted, fever manifestations were more common in DwoWS than in the DwWS and SD groups (59.3% versus 29.1% and 38.1%, *p* < 0.001). In contrast, days of fever and days of hospital stay in the DwWS and SD groups were longer than in the DwoWS group. Compared with the groups of DwoWS and DwWS, patients with SD had a greater proportion of warning signs (bleeding 57.1% versus 47.8% and 1.4%, *p* < 0.001, and hepatomegaly 23.8% versus 9.7% and 1.4%, *p* < 0.001). Abdominal pain was a more common symptom in patients with SD as compared with those of the DwoWS and DwWS groups (28.6% versus 6.0% and 3.6%, *p* < 0.001). A comparison of laboratory characteristics showed that patients with SD had higher mean levels of liver enzymes than patients with DwWS and DwoWS did (ALT 366.5 IU/l versus 54.5 IU/l and 35 IU/l, *p* < 0.001, and AST 1040 IU/l versus 104 IU/l and 55 IU/l, *p* < 0.001). There was no significant difference in renal function between the Dengue groups. Regarding hematological parameters, there was a significant decrease in platelet counts among the three Dengue groups. The lowest mean platelet counts were reported in patients with SD as compared with those in the DwoWS and DwWS groups (34 G/l versus 37.5 G/l and 100.5 G/l, *p* < 0.001). Conversely, patients with DwoWS and DwWS had lower WB counts than patients with SD (3.87 G/l and 3.73 G/l versus 7.21 G/l, *p* < 0.001). Among the 295 blood samples of patients suspected to have Dengue, Dengue serotypes were successfully detected in 223 samples. The distribution of Dengue serotypes according to Dengue severity is presented in Table 2. DENV-2 was predominant either as single or mixed infections. We also observed a higher proportion of DENV-2 in the SD group as compared to the DwoWS and DwWS groups, whereas DENV-1 had higher proportions in the DwoWS and DwWS groups (*p* = 0.032). Of note, DENV-4 and co-infection of DENV1-4 were only detected in the DwoWS and DwWS groups.

### 3.2. Levels of IL-18 and CXCL-10 Are Associated with Dengue Severity

**Figure 1:**
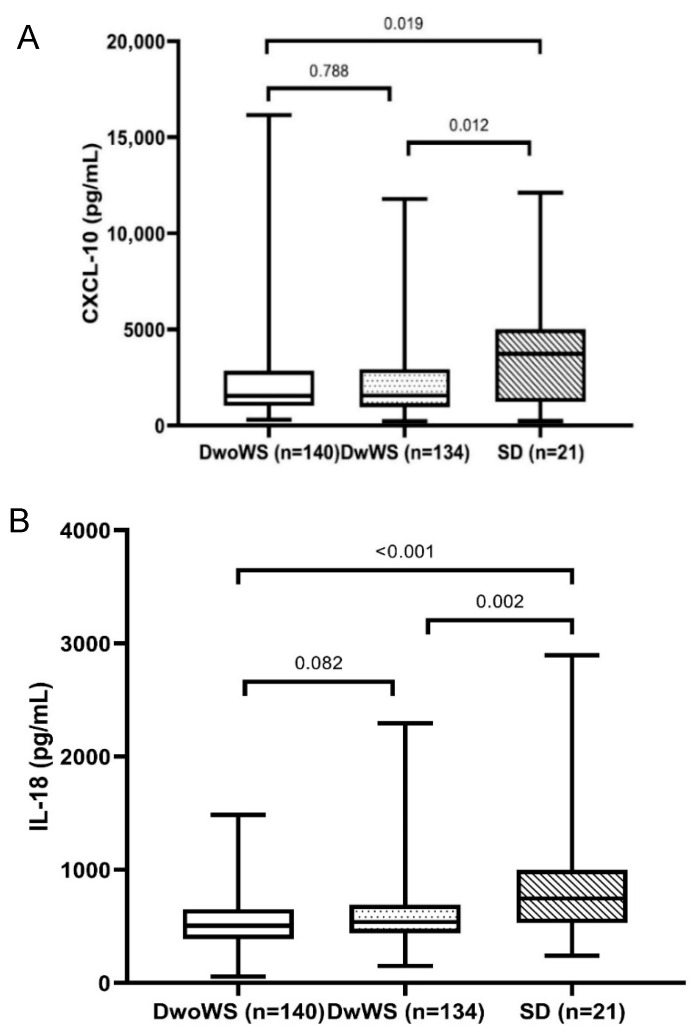
Comparison of CXCL-10 (**A**) and IL-18 (**B**) levels according to Dengue severity. Dengue without warning signs (DwoWS), Dengue with warning signs (DwWS), or severe Dengue (SD).

We measured and compared the levels of IL-18 and CXCL-10 with the different severity levels of Dengue. The results are presented in Table 2 and Figure 1. The mean levels of IL-18 pro-inflammatory cytokine in patients with SD were significantly higher as compared with those of the DwoWS (870.64 ± 550.93 pg/mL versus 546.26 ± 227.20 pg/mL, *p* < 0.001) and DwWS (870.64 ± 550.93 pg/mL versus 588.63 ± 247.78 pg/mL, *p* = 0.002) groups. However, there was no significant difference in IL-18 levels between the DwoWS and DwWS groups (588.63 ± 247.78 pg/mL versus 546.26 ± 227.20 pg/mL, *p* = 0.082). Similarly, significantly higher levels of CXCL-10 were detected in the plasma of patients with SD than in the DwoWS and DwWS groups (4013.48 ± 3554.50 pg/mL versus 2282.05 ± 2189.76 pg/mL, *p* = 0.019 and 2279.58 ± 2073 pg/mL, *p* = 0.012) (Table 3).

**Figure 2:**
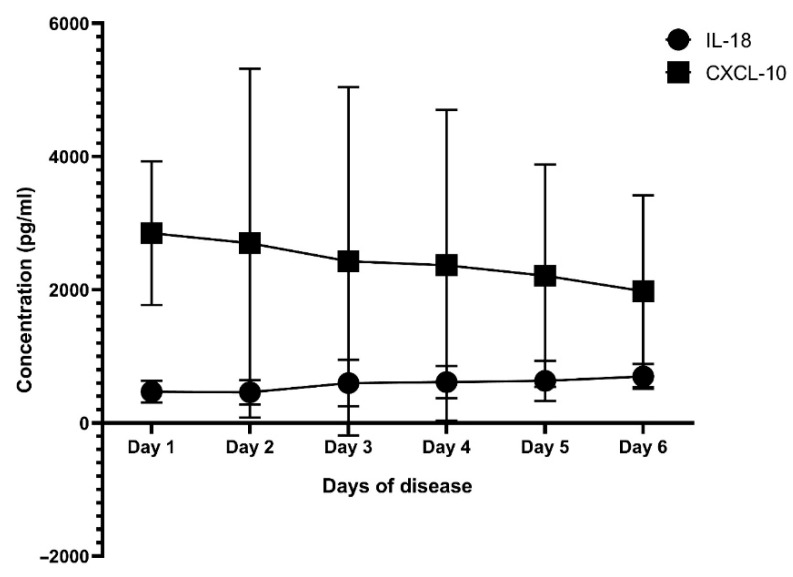
The mean levels of IL-18 and CXCL-10 at different days of disease. AST: aspartate aminotransferase; ALT: alanine aminotransferase.

**Figure 3:**
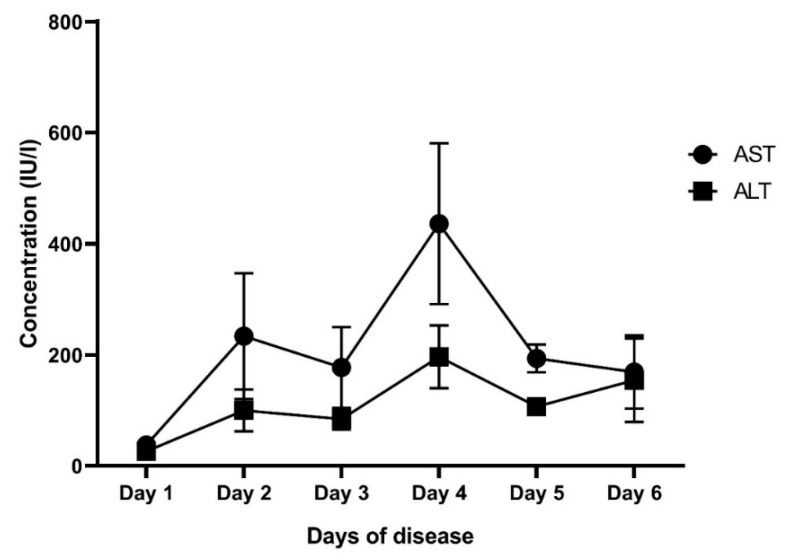
The mean levels of AST and ALT at different days of the disease. AST: Aspartate aminotransferase; ALT: Alanine aminotransferase.

Next, we analyzed the correlation of IL-18 and CXCL-10 with key laboratory parameters. The results are presented in Table 4. There was a positive correlation of IL-18 and CXCL-10 levels with liver enzymes in all patients, but when stratified in each group, we only observed the correlation of liver enzymes with IL-18, whereas CXCL-10 only had a positive correlation with AST levels in the DwoWS (r = 0.219; *p* = 0.016) and DwWS (r = 0.196; *p* = 0.03) groups, but there was no correlation in the SD group (*p* > 0.05). In contrast, platelet counts were negatively correlated with IL-18 levels in all patients (r = −0.361; *p* < 0.001), DwoWS (r = −0.448; *p* < 0.001), and DwWS (r = −0.216; *p* = 0.012). Of note, no correlation was found between CXCL-10 and key laboratory parameters in the SD group. Interestingly, when we analyzed the change in mean IL-18, CXCL-10, and liver enzyme levels according to the days of disease, we observed a consistent elevated IL-18 level during the course of disease, whereas CXCL-10 increased early in the febrile phase of the disease. In contrast, the elevation of liver enzymes tended to peak late in the course of disease (Figure 2 and Figure 3).

**Figure 4:**
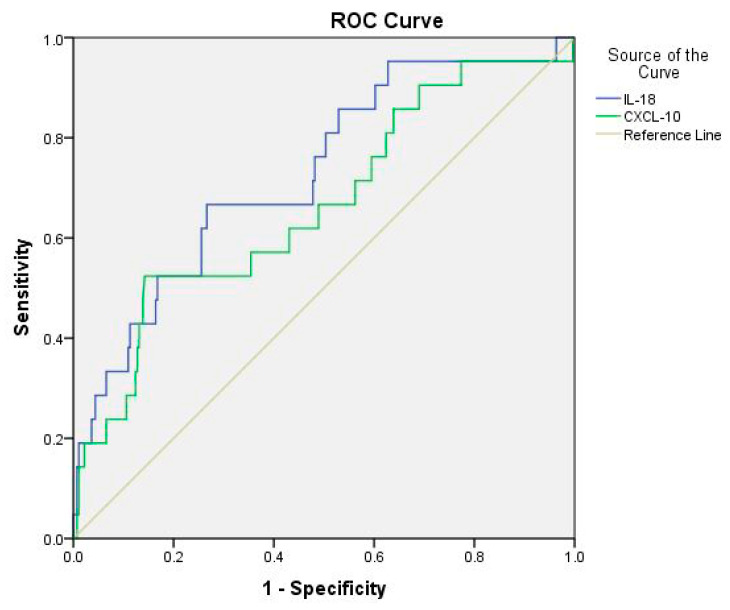
ROC curve analysis of IL-18 and CXCL-10 concentrations in predicting severe Dengue fever.

Furthermore, we analyzed the ROC curve to calculate cut-off values of IL-18 and CXCL-10 levels for predicting the severity of Dengue infection. The analysis revealed that the cut-off values of IL-18 were 669.65 (sensitivity: 66.7%; specificity: 73.4%; AUC: 0.729), and CXCL-10 levels were 3739.5 pg/mL (sensitivity: 52.4%; specificity: 85.8%; AUC: 0.665), which provided acceptable discrimination between DwoWS/DwWS and SD. Results are presented in Table 5 and Figure 4.

## 4. Discussions

In this study, we included 295 patients with clinical manifestations of Dengue infection in a tertiary hospital in Hanoi, Vietnam, and analyzed clinical and laboratory findings as related to disease severity. Vietnam is an endemic for Dengue, with an increasing incidence of Dengue reported in recent years, and Dengue infection occurs year-round, with the time of peak outbreak associated with the rainy season, from June to December, every year [11,12]. There have been endemic outbreaks of Dengue in Hanoi with the circulation of all four Dengue serotypes, but Dengue 1 and Dengue 2 are the most predominant serotypes [13,14,15]. In this study, based on the WHO’s 2009 classification [6], we showed that the proportion of severe Dengue was 7.11% (21/295). This is slightly higher than that observed in a previous study of patients admitted to a Referral Hospital in Hanoi [16] but lower than that reported by Huy et al., which showed a rate of 11.4% [12]. Of note, we found significant differences in clinical presentations and laboratory parameters between groups of patients—DwoWS, DwWS, and SD. Patients with SD exhibited a higher frequency of warning signs, including bleeding, hepatomegaly, and abdominal pain, as compared to those with DwoWS and DwWS. Similarly, levels of liver enzymes were elevated in patients with SD compared to those with DwoWS and DwWS (ALT 366.5 IU/l versus 54.5 IU/l and 35 IU/l, *p* < 0.001, and AST 1040 IU/l versus 104 IU/l and 55 IU/l, *p* < 0.001). These findings are consistent with previous studies [17], which explained that organ dysfunction and liver damage is common in Dengue infection and is recognized as a main indicator of SD [18]. In addition, abnormalities in hematological parameters showed a significant decline of platelet counts in the SD group, which resulted in an increased risk of bleeding and Dengue shock syndrome. Conversely, white blood cell (WB) counts were notably higher in patients with SD (7.21 G/l versus 3.87 G/l and 3.73 G/l in DwoWS and DwWS, respectively). Therefore, in clinical practice, marked indicators of routine blood tests during the course of Dengue infection could help to distinguish SD from non-SD. Of note, we showed a significant association of Dengue serotypes with disease severity, with a higher proportion of DENV-2 in SD (Table 2). Although all serotypes are the cause of Dengue disease with different manifestations, it has been found that the Asian strain of DENV-2 has a higher likelihood of causing severe disease than the American strain. This could be explained by the fact that the Asian strain of DENV-2 replicates more efficiently in human cells and can infect new mosquitos more effectively, resulting in increased transmission and a larger Dengue endemic outbreak [1,19].

Interestingly, in this study, we measured the levels of IL-18 and CXCL-10 and analyzed the potential association of these biomarkers with liver enzymes and disease severity. In fact, the role of IL-18, a pro-inflammatory cytokine associated with the Th1 immune response, has been implicated in various viral infections [20]. It has been shown that the interaction of virus and host factors results in the production and release of cytokine pro-inflammatory, anti-viral, and immunoregulatory cytokines that contribute to hepatic inflammation [3]. Importantly, the liver is an immune organ, and it plays a central role in the immune response to infections [21,22]. However, a hyperinflammatory response and overactive immune response trigger an excessive release of pro-inflammatory cytokines, which may contribute to vascular permeability, thereby contributing to the severity of the disease. In agreement with other studies [5,23], our study indicated that the mean level of IL-18 was significantly higher in patients with SD (870.64 ± 550.93 pg/mL) compared to the DwoWS (546.26 ± 227.20 pg/mL) and DwWS (588.63 ± 247.78 pg/mL) groups (*p* < 0.001). Furthermore, we also showed a positive correlation of IL-18 with liver enzymes in all patients, irrespective of severity (Table 4). It has been reported that high elevated liver enzymes are predictors of severe Dengue; however, these indicators appear later in the course of disease. In fact, progression to severe disease commonly occurs after the febrile phase (day 1 to 3), between days 4 and 6 of illness (critical phase). Hence, it was difficult to distinguish between SD and non-severe Dengue in the early stage of infection. In this study, we observed that the elevation of IL-18 levels seems to be early, while liver enzyme levels tend to peak late in the course of disease (Figure 2 and Figure 3). Therefore, the use of IL-18 could predict early disease progression to more severe Dengue. Although there have been several reports that used ROC analysis of AST and ALT to distinguish between severe Dengue and non-severe Dengue, providing the values of AUC in so doing, sensitivity and specificity were different [24,25,26]. This finding suggests that IL-18 may be a potential biomarker for disease progression and liver involvement in Dengue infection. Similarly, CXCL-10, a chemokine, plays an important role in attracting immune cells to sites of infection and immune activation and may thereby contribute to a cascade of cytokine production in Dengue infection [27]. It has been reported in various studies that elevation of IP-10 levels is clearly associated with plasma leakage and liver injury, especially in secondary Dengue infection [5,28,29]. However, CXCL-10 inhibits DENV replication by binding to heparan sulfate on the host membrane, promoting viral clearance [30,31]. In this study, we observed that patients with SD had significantly higher levels of CXCL-10 than those with DwoWS and DwWS (Table 2 and Figure 1). In addition, we revealed a positive correlation of CXCL-10 with liver enzymes in all patients with Dengue. This finding indicates the influence of CXCL-10 on liver enzyme increase and is linked to an indicator of disease severity.

Furthermore, based on the analysis of ROC curves, we determined the cut-off values of IL-18 and CXCL-10 for predicting Dengue severity. The cut-off value of IL-18 was 669.65 pg/mL (sensitivity: 66.7%; specificity: 73.4%; AUC: 0.729), whereas CXCL-10 was 3739.5 pg/mL (sensitivity: 52.4%; specificity: 85.8%; AUC: 0.665) (Table 5 and Figure 4). To the best of our knowledge, there is little information about the role of IL-18 and CXCL-10 in relation to disease severity among Vietnamese patients with Dengue infection. We suggest that these biomarkers are able to support clinicians in early diagnoses of severe Dengue, potentially aiding them in making proper clinical decisions in the monitoring and management of Dengue infection. Further studies should explore the mechanistic role of IL-18 and CXCL10 in Dengue pathogenesis and their potential as therapeutic targets for managing severe cases of the disease.

The present study had several limitations. This was a single-center and cross-sectional design study. Blood samples of patients with Dengue were only collected at a time point of admission for measuring of IL-18 and CXCL-10; therefore, we did not monitor dynamic changes in IL-18 and CXCL-10 levels during the course of the disease. In addition, we did not determine the history status of Dengue infection, which could have led to a difference in results. Finally, the number of patients with SD was small. Therefore, prospective studies with a larger sample size and molecular characterization of the Dengue virus will be necessary to validate our results.

## 5. Conclusions

This study indicates that IL-18 and CXCL-10 levels are significantly higher in SD compared to DwoWS and DwWS and were associated with disease severity among Vietnamese patients with Dengue infection. These findings suggest that IL-18 and CXCL-10 may be biomarkers for disease progression and disease severity in Dengue infection.

## Figures and Tables

**Table 1:** Characteristics of patients with Dengue infection.

Characteristics	DwoWS (n = 140)	DwWS (n = 134)	SD (n = 21)	*p*
Age (years)	41.5 (26.5–63.0)	38.0 (26.0–54.0)	58.0 (32.0–70.0)	**0.011**
Gender (male/female)	72/68	80/57	11/10	0.495
Days of Disease (days)	3.0 (2.0–4.0)	4.0 (4.0–5.0)	4.0 (3.0–4.0)	**<0.001**
Days of Fever (days)	4.0 (3.0–4.0)	4.0 (3.0–5.0)	4.0 (3.0–5.0)	**<0.001**
Red Blood Cells, T/l (n = 295)	4.60 (4.23–4.95)	4.91 (4.53–5.36)	4.64 (4.23–6.01)	**<0.001**
Hemoglobin, g/l (n = 295)	139.0 (126.5–149.0)	145.0 (132.0–158.0)	148.0 (122.0–173.0)	**0.005**
Hematocrit, l/l (n = 295)	0.410 (0.374–0.438)	0.434 (0.390–0.467)	0.426 (0.366–0.500)	**0.006**
Platelets, G/l (n = 295)	100.5 (43.5–155.5)	37.5 (15.0–84.0)	34.0 (12.0–95.0)	**<0.001**
White Blood Cells, G/l (n = 295)	3.87 (2.76–5.40)	3.73 (2.70–5.26)	7.21 (4.89–9.31)	**<0.001**
Neutrophil, % (n = 295)	62.95 (49.95–75.45)	54.35 (42.90–64.90)	71.60 (63.30–74.90)	**<0.001**
Lymphocytes, % (n = 295)	22.5 (13.7–33.7)	28.5 (21.6–37.4)	16.0 (11.7–27.3)	**<0.001**
Monocytes % (n = 295)	12.45 (9.25–16.90)	12.75 (9.30–17.50)	10.10 (8.50–13.70)	0.143
Creatinine, µmol/l (n = 267)	80.0 (60.0–98.0)	79.0 (64.0–94.0)	98.0 (71.0–147.5)	0.093
Urea, mmol/l (n = 250)	3.95 (3.10–5.40)	3.90 (3.10–5.10)	4.70 (3.60–13.80)	0.123
AST, UI/l (n = 264)	55.0 (29.0–98.0)	104.0 (47.0–172.0)	1040.0 (169.5–3934.5)	**<0.001**
ALT, UI/l (n = 265)	35.0 (23.0–60.0)	54.5 (33.0–90.5)	366.5 (70.0–1158.0)	**<0.001**
GGT, UI/l (n = 92)	53.5 (27.0–135.0)	107.0 (36.0–173.0)	120.0 (60.0–233.0)	**0.031**
Fever (n = 295)				
Yes, n (%)	**83 (59.3)**	39 (29.1)	8 (38.1)	**<0.001**
No, n (%)	57 (40.7)	**95 (70.9)**	**13 (61.9)**	
Body ache (n = 295)				
Yes, n (%)	119 (85.0)	105 (78.4)	19 (90.5)	0.212
No, n (%)	21 (15.0)	29 (21.6)	2 (9.5)	
Retro-orbital pain (n = 295)				
Yes, n (%)	8 (5.7)	7 (5.2)	2 (9.5)	0.734
No, n (%)	132 (94.3)	127 (94.8)	19 (90.5)	
Headache (n = 295)				
Yes, n (%)	92 (65.7)	75 (56.0)	12 (57.1)	0.208
No, n (%)	48 (34.3)	59 (44.0)	9 (42.9)	
Bleeding (n = 295)				
Yes, n (%)	2 (1.4)	**64 (47.8)**	**12 (57.1)**	**<0.001**
No, n (%)	138 (98.6)	70 (52.2)	9 (42.9)	
Vomiting/nausea (n = 295)				
Yes, n (%)	23 (16.4)	19 (14.2)	5 (23.8)	0.520
No, n (%)	117 (83.6)	115 (85.8)	16 (76.2)	
Anorexia (n = 295)				
Yes, n (%)	39 (27.9)	28 (20.9)	7 (33.3)	0.275
No, n (%)	101 (72.1)	106 (79.1)	14 (66.7)	
Diarrhea (n = 295)				
Yes, n (%)	22 (15.7)	16 (11.9)	3 (14.3)	0.664
No, n (%)	118 (84.3)	118 (88.1)	18 (85.7)	
Oliguria (n = 332)				
Yes, n (%)	6 (4.3)	8 (6.0)	**4 (19.0)**	**0.031**
No, n (%)	134 (95.7)	126 (94.0)	17 (81.0)	
Dyspnea (n = 295)				
Yes, n (%)	0 (0.0)	2 (1.5)	**17 (81.0)**	**<0.001**
No, n (%)	140 (100.0)	132 (98.5)	4 (19.0)	
Right Upper Abdominal pain (n = 295)				
Yes, n (%)	5 (3.6)	8 (6.0)	**6 (28.6)**	**<0.001**
No, n (%)	135 (96.4)	126 (94.0)	15 (71.4)	
Maculopapular rash (n = 295)				
Yes, n (%)	98 (70.0)	94 (70.1)	11 (52.4)	0.241
No, n (%)	42 (30.0)	40 (29.9)	10 (47.6)	
Hepatomegaly (n = 295)				
Yes, n (%)	2 (1.4)	13 (9.7)	5 (23.8)	**<0.001**
No, n (%)	138 (98.6)	121 (90.3)	16 (76.2)	
Splenomegaly (n = 295)				
Yes, n (%)	1 (0.7)	1 (0.7)	0 (0.0)	0.925
No, n (%)	139 (99.3)	133 (99.3)	21 (100.0)	

Abbreviations: DwoWS: Dengue without warning signs; DwWS: Dengue with warning signs; SD: severe Dengue; AST: aspartate aminotransferase; ALT: alanine aminotransferase; GGT: Gamma Glutamyl Transferase; DENV: Dengue virus. Data are represented as median (interquartile range) for continuous data or number with percentage for noncontinuous data. Chi-square/Fisher’s exact text, Kruskal–Wallis test/Mann–Whitney U tests where appropriate to compare among groups.

**Table 2:** Distribution of Dengue serotypes by Dengue severity.

Dengue Serotypes, n (%)	DwoWS	DwWS	SD	*p*
DENV-1	**29 (25.7)**	12 (12.8)	2 (12.5)	**0.032**
DENV-2	78 (69.0)	**81 (86.2)**	**13 (81.2)**	
DENV-4	1 (0.9)	1 (1.0)	0 (0.0)	
DENV1-2	1 (0.9)	0 (0.0)	1 (6.3)	
DENV1-4	4 (3.5)	0 (0.0)	0 (0.0)	
**Total (n = 223)**	**113 (50.7)**	**94 (42.2)**	**16 (7.2)**	

Abbreviations: DwoWS: Dengue without warning signs; DwWS: Dengue with warning signs; SD: severe Dengue; DENV: Dengue virus. *p* value was calculated by using the Chi-square test to compare among groups.

**Table 3:** Association between plasma IL-18 and CXCL-10 concentrations and Dengue severity.

Classification	IL-18 (pg/mL)		CXCL-10 (pg/mL)	
	Mean ± SD	Min–Max	Mean ± SD	Min–Max
**DwoWS (n = 140), (1)**	546.26 ± 227.20	55.80–1484.80	2282.05 ± 2189.76	292.30–16.160.00
**DwWS (n = 134), (2)**	588.63 ± 247.78	149.68–2295.10	2279.58 ± 2073.10	228.90–11.790.00
**SD (n = 21),** **(3)**	870.64 ± 550.93	242.00–2895.80	4013.48 ± 3554.50	236.50–12.120.00
p1-2	0.082		0.788	
p1-3	**<0.001**		**0.019**	
p2-3	**0.002**		**0.012**	

Abbreviations: DwoWS: Dengue without warning signs; DwWS: Dengue with warning signs; SD: severe Dengue; IL: interleukin; CXCL: C-X-C motif chemokine ligand; SD: standard deviation. Dengue virus. *p* value was calculated by using Mann–Whitney U tests to compare among groups.

**Table 4:** The correlation of IL-18 and CXCL-10 with laboratory parameters according to Dengue severity.

Groups	IL-18 (pg/mL)		CXCL-10 (pg/mL)	
	r	*p*	r	*p*
**All patients**				
RBC, T/l (n = 295)	**0.147**	**0.012**	**0.122**	**0.036**
Platelet, G/l (n = 295)	**−0.361**	**<0.001**	**−0.146**	**0.012**
AST, UI/l (n = 264)	**0.505**	**<0.001**	**0.245**	**<0.001**
ALT, UI/l (n = 265)	**0.484**	**<0.001**	**0.203**	**0.001**
WBC, G/l (n = 295)	0.052	0.375	−0.009	0.879
**DwoWS**				
RBC, T/l (n = 140)	0.154	0.069	0.138	0.103
Platelet, G/l (n = 140)	**−0.448**	**<0.001**	**−0.178**	**0.035**
AST, UI/l (n = 121)	**0.478**	**<0.001**	**0.219**	**0.016**
ALT, UI/l (n = 121)	**0.509**	**<0.001**	0.175	0.055
WBC, G/l (n = 140)	−0.141	0.097	−0.020	0.816
**DwWS**				
RBC, T/l (n = 134)	0.118	0.173	0.150	0.083
Platelet, G/l (n = 134)	**−0.216**	**0.012**	−0.078	0.373
AST, UI/l (n = 123)	**0.435**	**<0.001**	**0.196**	**0.030**
ALT, UI/l (n = 124)	**0.371**	**<0.001**	0.160	0.075
WBC, G/l (n = 134)	0.091	0.295	−0.075	0.391
**SD**				
RBC, T/l (n = 21)	−0.126	0.586	−0.211	0.360
Platelet, G/l (n = 21)	−0.227	0.322	−0.344	0.127
AST, UI/l (n = 20)	**0.529**	**0.016**	−0.015	0.950
ALT, UI/l (n = 20)	**0.499**	**0.025**	−0.022	0.927
WBC, G/l (n = 21)	0.390	0.081	−0.345	0.125

Abbreviations: DwoWS: Dengue without warning signs; DwWS: Dengue with warning signs; SD: severe Dengue; AST: aspartate aminotransferase; ALT: alanine aminotransferase; RBC: red blood cells; WBC: white blood cells. r and *p* values were calculated by Spearman’s rank correlation coefficient.

**Table 5:** AUC values obtained from ROC analysis for IL-18 and CXCL-10 associated with Dengue severity.

Tests	AUC	Cut-off	Se (%)	Sp (%)
IL-18 (pg/mL)	0.729	669.65	66.7	73.4
CXCL-10 (pg/mL)	0.665	3739.5	52.4	85.8

Abbreviations: AUC, area under the curve; ROC, receiver operating characteristic; IL-18: interleukine-18; CXCL-10: C-X-C motif chemokine ligand 10.

## Data Availability

Data are available upon reasonable request.

## Abbreviations

The following abbreviations are used in this manuscript:

IL-18	Interleukin 18
CXCL-10	chemokines C-X-C motif ligand-10
WHO	World Health Organization
